# Clinical Efficacy and Tolerability of a New Experimental Mucoadhesive Patch for Topical Anesthesia of Oral Mucosa in Pediatric Dentistry

**DOI:** 10.3390/jcm13061558

**Published:** 2024-03-08

**Authors:** Gianmaria Fabrizio Ferrazzano, Giuseppe Di Fabio, Sara Caruso, Roberto Gatto, Varinder Goyal, Silvia Caruso

**Affiliations:** 1 UNESCO Chair in Health Education and Sustainable Development, Paediatric Dentistry Section, University of Naples “Federico II”, 80138 Naples, Italy; 2 Department of Life, Health and Environmental Sciences, Paediatric Dentistry, University of L’Aquila, 67100 L’Aquila, Italy; saracaruso2704@gmail.com (S.C.); roberto.gatto@univaq.it (R.G.); silvia.caruso@univaq.it (S.C.); 3 Department of Paediatric & Preventive Dentistry, Guru Nanak Dev Dental College & Research Institute, Sunam 148028, Punjab, India; virinderg@gmail.com

**Keywords:** dental injection, lidocaine, pain management, pediatric dentistry, prilocaine, topical anesthetic, topical patch

## Abstract

**Background**: The injection of local anesthetics is the procedure that still causes the most fear and anxiety in a dental session; to minimize this problem, we can use topical anesthesia. The aim of this study is to analyze the tolerability and the clinical efficacy in the control of pain, during the subsequent injection of the local anesthetic, of an experimental anesthetic patch with a new formulation, which was previously tested in gel formula. **Methods**: A total of 150 children, aged 4 to 9 years, were included in the study. Each patient was treated using three different pre-anesthesia methods (placebo gel, experimental gel, and experimental patch), according to a split-mouth procedure, within a week of each other. The injection pain was analyzed using the WBFPRS and FLACC scales. Patients’ caregivers’ satisfaction was recorded at the end of the procedures. The data were analyzed using one-way ANOVA-RM, Wilcoxon–Mann–Whitney, Mann–Whitney U, and χ^2^ tests. **Results**: In this study, significantly higher pain ratings were observed with the topical placebo gel and lower pain ratings were observed with the experimental patch. **Conclusion**: The use of the patch proved to be very effective in reducing pain both subjectively and objectively, in the absence of both local and systemic side effects, validating its use in the oral mucosa.

## 1. Introduction

The term “anesthesia” refers generically to the loss of sensations, mainly sensitivity and pain. Anesthesia-inducing drugs, called anesthetics, are used during surgery or other medical procedures to numb certain parts of the body or to generate an artificial sleep state. The use of anesthetics prevents the perception of pain and other unpleasant sensations, allowing the execution of a wide range of medical and surgical procedures. Anesthetics are commonly divided into two classes: local anesthetics and general anesthetics [1].

Local anesthetics, used for minor procedures or interventions, cause loss of sensation in a limited area of the body, maintaining the state of consciousness and temporarily blocking only the nerve fibers that carry the sensation of pain [2]. As soon as the effect of the anesthetics wears off, nerve impulses can reach the brain again, allowing sensation to resume.

Pain is a sensation that has often been associated with dental care [3]. There are many treatments in daily dental clinic practices that, without proper anesthesia, would lead to painful sensations. For example, even the treatment of caries, the most common chronic pathology in the world in both children and adults [4] and, together with orthodontic treatments [5], the most performed procedure in the pediatric population, would be impossible to complete without anesthesia.

From the national and global prevalence data [6,7], it is clear how necessary it is to develop a local anesthesia that causes the least trauma possible, since, especially in the pediatric population, any unpleasant sensation could lead to a loss of patient compliance and, in the worst cases, to a failure in completing the treatments, leaving the problem of oral health in the young patients [8].

A local or regional injection anesthesia is essential to eliminate or minimize the pain caused by dental treatments. However, needle-phobia is among the main factors that cause patients anxiety, to the point of delaying or canceling treatments, significantly compromising their oral-health-related quality of life [9,10], or arriving at situations in which it is necessary to carry out the treatment either under deep sedation [11], which is associated with local anesthesia, or even under general anesthesia [12], similarly to what occurs when the patient suffers from disabilities or other systemic pathologies.

In fact, dental fear or dental anxiety is a large problem in children/adolescents worldwide [13]. Therefore, it is essential to build the best possible doctor–patient relationship, constantly use reassuring language, and always maintain good pain control to obtain good compliance that allows us to complete all the necessary dental treatments. So, the use of topical anesthesia, to be used before performing the injection of the local anesthetic, helps us to eliminate the needle-phobia.

Topical anesthesia produces a temporary and superficial loss of sensitivity in a limited area: the anesthetic effect is in fact obtained with direct application on the intervention area. This would eliminate the pain during needle penetration and local anesthetic emission, which leads to the negative sensations associated with an injection. Furthermore, the mucous membranes, such as the mouth, genitals, and conjunctiva, are more easily penetrated by topical anesthetics than through a keratinized surface because of the absence of a stratum corneum. To facilitate the passage of the topical anesthetic through the mucosa, the latter must be as dry as possible, the formulation of the anesthetic must have higher concentrations than the injection formulations, and no vasoconstrictors should be added.

Among the various molecules discovered, the ones used most as topical anesthetics are the following [14]. Lidocaine is the most used local anesthetic, and it has an intermediate toxicity and potency, a high affinity for fat tissues, and a high solubility in water [15]. Prilocaine has a high tolerability and has no vasodilatory action; therefore, it persists longer in the tissues. Benzocaine requires high concentrations, ranging from 10 to 20%, to have an anesthetic effect and has low solubility. Alternatively, eutectic mixtures of local anesthesia (EMLA) can be used, in which multiple anesthetic molecules are combined to improve their characteristics [16]. Eutectic mixtures are compounds (5% oil in water emulsion cream with 25 mg/mL of lidocaine, 25 mg/mL of prilocaine) that melt at lower temperatures than any of their components, permitting higher concentrations of anesthetics for use.

Currently, the versatility of topical preparations allows their use in various branches of medicine, including ophthalmology, dermatology, and dentistry. In dentistry in particular, the topical anesthesia can be used before the injection or for other simple operations such as the extraction of a deciduous tooth or the positioning of a rubber dam clamp.

Anesthetic molecules can have some adverse reactions specific to certain routes of administration. In particular, in local anesthesia, the excessive absorption of the drug, or inadvertent intravascular administration, can result in LAST (local anesthetic systemic toxicity). The presentation of LAST has possible variants. Classically, toxicity appears on a continuum of adverse effects in the CNS (dizziness, drowsiness, tinnitus, confusion, dysphoria, dysarthria, auditory disturbances, seizures, loss of consciousness, agitation) that, with more toxic levels, progresses to cardiovascular symptoms (hypertension, tachycardia, ventricular arrhythmias, cardiogenic shock, and refractory hypotension). Among the other side effects of anesthetic molecules, we include allergies and methemoglobinemia (with prilocaine [17] and benzocaine [18]). So, one of the most important advantages of the topical formulation is low significant systemic drug absorption, which may reduce the risk of systemic adverse effects and drug–drug interactions [19]. Indeed, regarding methemoglobinemia, for example, the cases associated with topical gel are rather rare, and topical molecules are contraindicated only in cases of the concomitant use of a methemoglobin-inducing agent, such as nitrates or aniline, or infants less than three months old or premature [20].

Topical medication can be given in different ways: spray, gels, solutions, ointments, and patches. Patches in particular have high mucoadhesive properties and strong adhesion occurs when saliva is not present at the application site, leading to rapid and boosted drug release.

Therefore, the aim of this study was to test the clinical efficacy and tolerability of an experimental anesthetic patch with a new formulation (10% lidocaine, 10% prilocaine) to analyze the efficacy and potency in the control of pain during the subsequent injection of local anesthetic and the presence of any side effects. To validate the potency of the patch formulation, it was compared to two different control groups: placebo and the same anesthetic composition (10% lidocaine, 10% prilocaine) in a gel formula that was already previously tested in another study [21]. All three procedures were tested on the same patient, according to a split-mouth procedure.

## 2. Materials and Methods

### 2.1. Data Source

The experimental protocol was approved by the Research and Ethics Committee of the University of Naples “Federico II” (Prot. N. 23/2019). At the beginning of this study, conducted from January 2022 to March 2023, 170 children, aged 4–9 years, were recruited. A minimum sample size of 127 subjects was determined via the G*Power software program (power = 0.80, α = 0.05, β = 0.20. G*Power Version: 3.1.9.2; Heinrich-Heine-Universität Düsseldorf, Düsseldorf, Germany).

The operations were conducted in the Pediatric Dentistry Center in Sedation of Naples and supported by the UNESCO Chair in Health Education and Sustainable Development of “Federico II” University of Naples between February 2022 and February 2023 by six pediatric dentists. All examiners were calibrated on pain recording at the UNESCO Chair of Naples, and the kappa test revealed a final score of k = 0.90 (CI 95% 0.785–0.941).

All patients had never undergone infiltrative anesthesia and were American Society of Anesthesiology (ASA) Type I, as defined by the ASA Physical Status Classification System. Patients who needed multiple treatments that required anesthesia were selected. Therefore, patients who presented pain, inflammatory, or infectious processes (such as irreversible pulpits or abscesses) at the time of treatment were excluded. Among the exclusion criteria based on medical history, there was also a history of allergies, the presence of chronic pathologies, and the use of medications (such as NSAIDs or corticosteroids) at the time of treatment.

After exclusion, the final sample size was 150 children.

Written informed consent, with an explanation of potential risks and benefits, was discussed and obtained from each caregiver of all eligible children who agreed to participate.

### 2.2. Individual Variables

In this clinical research, each patient was treated with 3 different pre-anesthesia methods, according to a split-mouth procedure, within a week of each other:

Method A (placebo gel): In the first treatment session, the injection site was dried out, and strawberry-flavored placebo gel, with the same appearance and viscosity as topical gel, was applied with a cotton pellet, using moderate pressure with rubbing circular motion for 30–45 s to a confined site and left for about 5 min.

Method B (topical anesthetic gel): In the second treatment session, the injection site was dried out, and strawberry-flavored topical anesthetic gel, containing 10% lidocaine, 10% prilocaine, and mucoadhesive gel q.b., was applied with a cotton pellet, using moderate pressure with rubbing circular motion for 30–45 s to a confined site and left for about 5 min.

**Figure 1:**
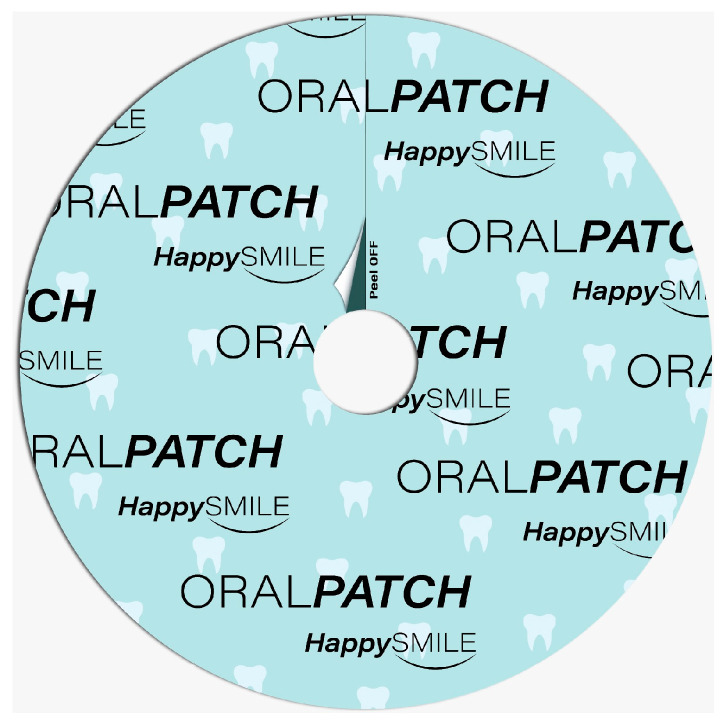
A representation of the experimental patch.

Method C (anesthetic patch group): In the third treatment session, a strawberry-flavored 2 × 2 cm patch (Figure 1) was prepared with a single waterproof polymer layer, loaded with a topical anesthetic gel, containing 10% lidocaine, 10% prilocaine and mucoadhesive gel q.b.; after the injection site was dried out, the patch was customized (cut to best fit the site to be anesthetized), applied for 3 min, and then removed before local injection.

The patients, after each pre-anesthesia method, received an injection of an LA solution (Ultracain D-S forte; Hoechst Cana-da 145 Inc., Montreal, QC, Canada) using a 27-gauge dental needle, as follows: the patient opened their mouth, and, with the use of reassuring language, the patient’s lip was lifted keeping the tissue taut; the needle was inserted parallel to the long axis of the tooth at the level of the mandibular or maxillary fornix, always on the posterior teeth; the injection was carried out at a flow rate of 1 mL/minute, initially by injecting the anesthetic into the more superficial mucosal layers, after which the introduction of anesthetic into the deeper layers was continued; and, finally, the needle was gently removed.

The effectiveness of pre-anesthesia methods with respect to injection pain was assessed subjectively and objectively, and the patients’ caregivers were asked about their assessment.

**Figure 2:**
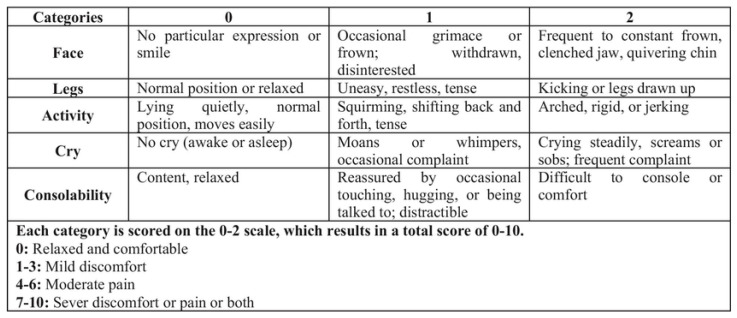
The Face, Legs, Activity, Cry, and Consolability (FLACC) behavioral pain assessment scale.

Objective assessment: The FLACC scale was used for objective assessment [22] (Figure 2). It belongs to the multidimensional pain measurement scales based on the observation of the child’s behavior. The maximum score is 10 and represents the maximum expression of pain; the minimum is 0 and represents the absence of pain.

**Figure 3:**
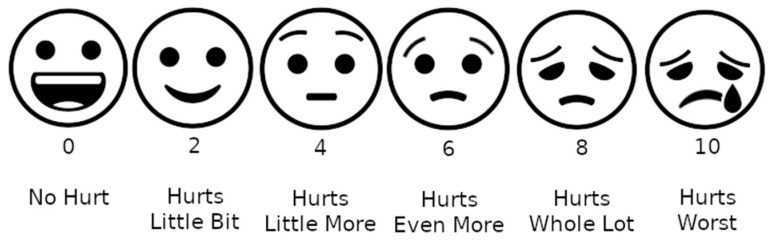
A representation of the Wong–Baker scale.

Subjective assessment: The WBFRRS, or Wong–Baker Faces PRS (Pain Rating scale) was used for subjective assessment [23] (Figure 3). It is a one-dimensional pain rating scale used for children between 3 and 8 years of age. The scale shows a series of faces ranging from a happy face at 0, or “no pain”, to a crying face at 10, which represents “the worst pain imaginable”. Based on the faces and written descriptions, the patient chooses the face that best describes their level of pain.

Patients’ caregivers’ assessment: at the end of the collection of pain scale data, the satisfaction rate of the caregivers, present throughout the procedure, was also recorded, through a simple anonymous response in written form.

For each patient, there number of pediatric dentists working simultaneously was three: one operator administered the pre-anesthesia method, one operator administered the local anesthetic, and a third collected the data regarding the pain scales. The operator who performed the local anesthesia and the one who collected the data were not aware of the pre-anesthetic method used.

### 2.3. Statistical Analysis

The data obtained were statistically analyzed with Stata software (Version: 14.1; Stata Corp LP, College Station, TX, USA). The analysis of variance–repeated-measures (one-way ANOVA-RM) was used to determine the significance of the difference in pain scores between the 3 groups, after a logarithmic transformation for normalization of the data. When the resulting differences were statistically significant, post hoc analysis was performed using the Wilcoxon-Mann–Whitney test for pairwise comparisons. The relationship between pain ratings and gender was evaluated with the Mann–Whitney U Test. The χ^2^ test was used to compare the distribution of caregivers’ patient assessment. The significance level (α) was set to 0.05. Only patients with complete data on all analyzed variables were included in the analysis.

## 3. Results

The trial was completed with good compliance. No allergic reactions or side effects were observed. As shown in Table 1, 150 children, 81 girls (54%) and 69 boys (46%), aged between 4 and 9 years (6.54 ± 0.85) were included in this study. The relationship between pain ratings and gender, age, and injection’s site (maxillary or mandibular) is not included in the tables, as the statistical tests showed that there are no statistically significant differences (*p* > 0.05) in both the PRS and FLACC scales.

**Figure 4:**
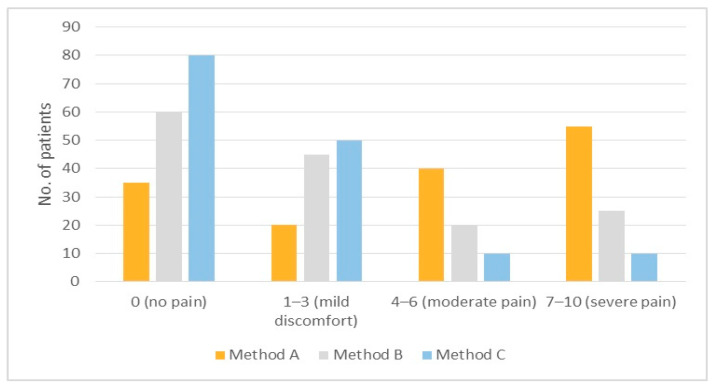
FLACC scores with Methods A, B, and C.

**Figure 5:**
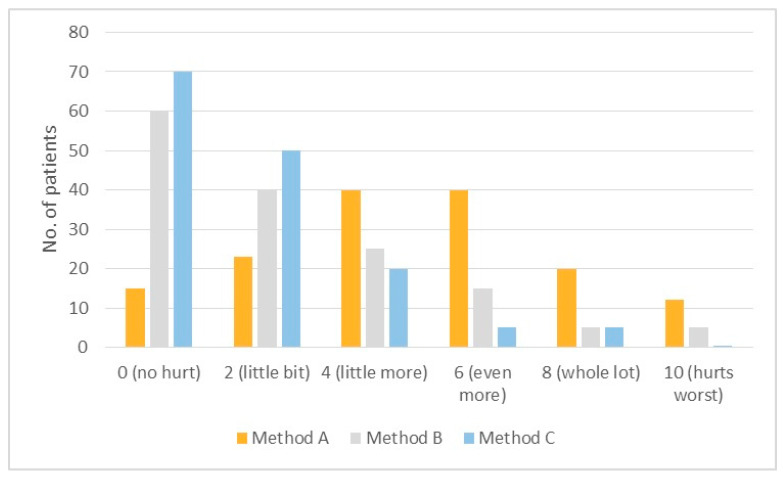
PRS scores with Methods A, B, and C.

Both pain rating scales, PRS (*p* < 0.001) and FLACC (*p* < 0.001), showed statistically significant differences between the three groups (Table 2 and Table 3, Figure 4 and Figure 5). In particular, in both tables, Method C (Patch group) showed lower pain ratings.

On the FLACC scale, the mean score of Method C (1.56 ± 0.21) was better in terms of pain control than that of Method B (2.68 ± 0.45) and Method A (4.71 ± 0.63). In fact, the number of “no pain” ratings was higher with Method C than in the other groups (C: 80; B: 60; A: 35). In addition, the number of “severe pain” ratings was lower with Method C than with Method B and A (C: 10; B: 25; A: 55).

Likewise, on the Wong–Baker PRS scale, the mean score of Method C (1.42 ± 0.28) was lower than that of Method B (2.12 ± 0.72) and Method A (4.39 ± 1.25). More precisely, the number of “no pain” ratings was higher with Method C than with Method B and A (C: 70; B: 60; A: 15), and the number of “worst pain” ratings was lower with Method C than in the other groups (C: 0; B: 5; A: 12).

There was also a statistically significant difference in terms of caregivers’ patient assessments, according to the χ^2^ test (*p* < 0.001). As shown in Table 4, with Method C, the results were the highest with almost all (97%) of caregivers satisfied with the procedure; with Method B, the results were also positive with the majority; while alternatively, with Method A, the percentage satisfied with the procedure decreased to 43%.

## 4. Discussion

The aim of the study was to evaluate the use of an anesthetic patch with the experimental formulation in the control of pain at the time of injection.

In fact, the administration of anesthesia for dental treatments is still necessarily of the injection type, despite many innovations having been made in other fields of dentistry [24], and unfortunately, the injection itself remains the source of most anxiety in dental sessions. As a result, some patients during certain dental procedures preferred mild or moderate pain rather than receiving an injection [25].

Therefore, in clinical dental practice, a pre-anesthesia technique is often necessary, especially in the pediatric population, so topical anesthesia comes to our aid. Even today, too often, children do not undergo dental treatment for fear of pain, or they may have exaggerated reactions and completely lose trust in the dentist, not allowing the treatment to be completed; therefore, it is necessary to avoid any discomfort, including that of injecting the anesthetic. Therefore, this study was required because it appeared necessary to find a simple topical anesthesia method that is easy to manage intraoperatively and that can make the dental session totally pain-free, a topic that is currently underestimated in scientific literature.

Indeed, despite the help that topical anesthesia could give us, and although the literature confirmed the validity of topical anesthesia as a pre-anesthetic method in dentistry, studies on topical anesthetics focused on the gel formula were often inconsistent and tended to compare the various molecules (usually EMLA or other mixtures and 20% benzocaine) with conflicting results. Milani et al. [26] and Abu et al. [27] found that EMLA (2.5% lidocaine and 2.5% prilocaine) was significantly better in controlling pain than 20% benzocaine before injection into the maxilla. Reznik et al. [28] confirmed that another mixture containing 20% lidocaine, 4% tetracaine, and 2% phenylephrine performed better on pain scales than 20% benzocaine in reducing pain during TADs placement. Meanwhile, according to Primosch and Rolland [29], EMLA and benzocaine at 20% were comparable in reducing pain for injections into the palatal mucosa in pediatric patients. Differently, Tulga and Mutlu [30] stated that EMLA performed worse than benzocaine 20% as a pre-anesthesia gel in a group of pediatric patients.

Therefore, in dentistry, studies regarding topical anesthesia were present, but they are often conflicting and inconclusive; instead, there were few studies that focused on anesthetic patches. In general, medicine patches of 5% lidocaine are often used in the treatment of postherpetic neuralgia [31], in which this type of patch was also applied 18/24 h a day for three days without major side effects and with a low systemic absorption of the molecule [32]. These data are also confirmed with the new formulation since, in our sample studied, no adverse events or undesirable effects occurred after the application of the patch, certifying its safety and tolerability both locally and systemically.

While the great tolerability of this lidocaine–prilocaine patch is a welcome improvement, the more important clinical consideration is whether it controls pain.

Regarding the few studies in dentistry that have been carried out to evaluate the application of patches on the oral mucosa, none of these have ever used this experimental mixture of anesthetics, but the most-used anesthetics have been patches with 10% or 20% lidocaine [15,33]. Instead, the decision was made to use a mix of lidocaine and prilocaine in this experimental patch, choosing prilocaine for its speed of action; in fact, the patch was kept in place in 3 min, compared to previous studies, where it was left for 5 to 15 min. This is because in the pediatric population, when the treatment is prolonged excessively, there is a risk of losing the child’s compliance; therefore, a topical anesthesia technique is possibly needed with a certain rapidity of action.

Then, the evaluation of the clinical efficacy of the patch was carried out, compared to the experimental gel and the placebo gel, obtaining the best results for pain control in both subjective and objective scales, with about 80% of children who had almost no pain with the injection, which makes treatment much easier to follow. This efficacy is probably attributable to the great adherence of the patch to the underlying mucosa, which allows the maximum concentration of the topical anesthetic to be absorbed, once the mucosa is dried and the patch is applied, without the washing effect of the saliva, which could compromise its effectiveness, as happened with the gels. Also, as a side observation, although the gels had a pleasant taste, as far as the strawberry aroma was concerned, good aspiration control was still needed to prevent saliva from spreading the gels into unwanted areas, which could annoy the subjects. In fact, a topical anesthetic that stays in place and prevents unpleasant taste is preferable for both the patient and the dentist, characteristics that the experimental patch evidenced thanks to the excellent muco-adhesiveness and the small size, which allowed its excellent adaptability in every area of the buccal mucosa, compared to other patches studied, in which the major defects were precisely the size and the poor adhesiveness [16].

Another important point to be underlined is the choice to use the three methods sequentially. Regarding this point, a precise evaluation was made: a fast pilot study (about 30 patients) was conducted in which the sequence of the three procedures was randomized, and the patch gave the best results; therefore, in the main study, the objective was to evaluate whether these excellent results were also confirmed in a situation in which the subject could have had possible negative experiences in previous sessions. So, from the results of this study, the previous experience, in the subjects who experienced the most annoyance, did not influence the results of the new anesthetic formulation, mostly in the patch, in which excellent pain control was obtained, especially since it was the third appointment; this success meant that even the satisfaction of the children’s caregivers gradually improved.

The limitation of this study is the absence of information concerning the determination of lidocaine–prilocaine plasma concentrations in the subjects, which would be useful for validating the greater absorption of the molecules in the patch compared to the gel. This analysis was avoided so as not to subject these pediatric patients to a bothersome procedure such as blood sampling.

Pain control with the local anesthetic represents a fundamental first step in the pediatric dental treatment. The patient is capable both of laying the foundations for excellent compliance in the procedures in the event of no discomfort, and of compromising the future relationship between doctor and patient in the case of pain. In this study, we tested this new formulation of a topical anesthetic, evaluating the main pain scales as parameters. From the data obtained, there are excellent values in pain reduction for both of these types of topical anesthetics; however, the best results were achieved when using the anesthetic patch, and, in addition, there was a total absence of side effects. Further studies could compare its use as an alternative to local anesthesia to evaluate its effect alone in pain control in some oral treatments, especially those concerning the mucous layers, such as subgingival scaling or gingivoplasty.

## 5. Conclusions

The use of a patch, proved to be very effective in reducing pain both subjectively and objectively, surpassing both the gel with the experimental formulation and the placebo gel. It has also demonstrated its anesthetic capabilities with the absence of both local and systemic side effects, validating its use in the oral mucosa. Therefore, the use of a topical anesthetic represents a tool that modern pediatric dentistry cannot afford to exclude, to decrease discomfort and increase clinical compliance.

## Figures and Tables

**Table 1:** Demographics and clinical characteristics of the study population.

Variable		Study Population (No. 150)
Age	4 years	15 (10%)
	5 years	21 (14%)
	6 years	36 (24%)
	7 years	41 (27%)
	8 years	20 (14%)
	9 years	17 (11%)
Gender	Male	69 (46%)
	Female	81 (54%)
ASA class I		150 (100%)

**Table 2:** Distribution of the FLACC scale pain ratings.

FLACC Scores						
	No.	0	1–3	4–6	7–10	Mean (SD)
Method A	150	35 (23%)	20 (13%)	40 (27%)	55 (37%)	4.71 ± 0.63
Method B	150	60 (40%)	45 (30%)	20 (13%)	25 (17%)	2.68 ± 0.45
Method C	150	80 (53%)	50 (33%)	10 (7%)	10 (7%)	1.56 ± 0.21

*p* < 0.001 statistically significant with Student’s *T* test.

**Table 3:** Distribution of the PRS scale pain ratings.

PRS Scores								
	No.	0	2	4	6	8	10	Mean (SD)
Method A	150	15 (10%)	23 (15%)	40 (27%)	40 (27%)	20 (13%)	12 (8%)	4.39 ± 1.25
Method B	150	60 (40%)	40 (27%)	25 (17%)	15 (10%)	5 (3%)	5 (3%)	2.12 ± 0.72
Method C	150	70 (47%)	50 (33%)	20 (13%)	5 (3%)	5 (3%)	0 (0%)	1.42 ± 0.28

*p* < 0.001 statistically significant with Student’s *T* test.

**Table 4:** Distribution of caregivers’ patient assessment.

Caregivers’ Patient Assessment			
	No.	Satisfied	Not-Satisfied
Method A	150	65 (43%)	85 (57%)
Method B	150	130 (87%)	20 (13%)
Method C	150	145 (97%)	5 (3%)

*p* < 0.001 statistically significant with Fisher’s exact test.

## Data Availability

Data will be made available upon reasonable request to the authors.
